# The clinical outcomes and complications of combined fixation with cannulated screws and the modified Pyrford technique for the treatment of transverse patellar fractures: a case series study

**DOI:** 10.1186/s12893-022-01788-5

**Published:** 2022-09-10

**Authors:** Yihan Li, Qingxian Tian, Kunpeng Leng, Meng Guo

**Affiliations:** Orthopedic Department of Beijing Chaoyang Hospital, Capital Medical University, Gongti South Road 8, Chaoyang District, Beijing, 100020 China

**Keywords:** Transverse patellar fractures, Cannulated screws, Nonabsorbable polyester sutures, Complications, Clinical outcomes, Device removal

## Abstract

**Background:**

Transverse patellar fractures can be fixed using various techniques. The purpose of the current study was to assess the clinical outcomes and complication rate of a combined fixation technique using cannulated screws and the modified Pyrford technique with nonabsorbable polyester sutures.

**Methods and patients:**

Between January 2015 and February 2021, 26 transverse patellar fractures were fixed with this combined technique. Preoperative data were collected from patients with transverse patellar fractures who were followed up for at least 12 months. At each follow-up visit, plain radiographs were taken. At the 12-month postoperative follow-up, range of motion of the affected knee joint and clinical outcomes, as evaluated by the Bostman scoring system, were recorded.

**Results:**

The average Bostman score at the 12-month postoperative follow-up was 28.3 ± 1.5. Furthermore, the average extension and flexion of the knee joint were 1.2 ± 2.1 and 125.6 ± 6.7 degrees, respectively. One patient experienced delayed bone union and one experienced superficial wound infection. There were no other postoperative complications. One patient required removal of the device for social-psychological reasons.

**Conclusions:**

The combined fixation technique with cannulated screws and the modified Pyrford technique with suture materials produced excellent clinical outcomes and a low rate of complications in the treatment of transverse patellar fractures.

## Background

Patellar fractures constitute approximately 1% of all adult skeletal fractures [1]. The patella is a key point of the extensor apparatus, which transports the contraction force that originates in the quadriceps to the tubercle of the tibia to achieve extension of the knee joint. Furthermore, the patella increases the moment arm of the quadriceps tendon by 30% to facilitate extension of the joint [2]. Interruption of the extensor apparatus due to a patellar fracture necessitates surgical fixation due to the key role of the patella in the extensor apparatus. Surgical fixation is required to achieve anatomic reduction and stable fixation, allowing for early rehabilitation and a lower rate of posttraumatic osteoarthritis [3].

The modified tension band was composed of a looped figure-eight metallic tension band wire and double K-wires [4]. Although this technique can be applied in any type of patellar fracture, the rate of postoperative complications after the modified tension band technique ranges from 21–53% [5–7]. Complications include Kirschner wire migration, tension band breakage, fixation failure, pain, symptomatic hardware, and infection [5–7]. Biomechanically, transverse patellar fractures fixed with parallel cannulated lag screws and a metallic wire are more stable [8]. However, secondary surgeries to remove the internal fixation device are required in 23% of patients who undergo this surgical treatment [9]. To avoid the high rate of internal fixation device removal, a variety of patellar cerclage or tension band fixation techniques with suture materials, in place of a metallic device, have been introduced over the past several decades; these have dramatically decreased the postoperative device removal rate to 0 [10–13]. However, the failure rate after fixation involving patellar cerclage or tension bands with suture materials is reported to range from 6.3 to 7.7% [10, 13]. Illical et al. [7] fixed patellar fractures with parallel Kirscher wires and a tension band with Ethibond sutures. They found that none of the patients required secondary surgery due to failure of fixation or bone non-union; however, the postoperative complication rate was as high as 45.5% [7]. The Pyrford technique is a technique for treating patellar fractures that involves fixation with a metallic circumferential cerclage wire and a metallic anterior tension band [14]. Camarda et al. [12] modified this technique by substituting the metallic wire with suture material for the treatment of patellar fractures. The authors reported good clinical outcomes and high postoperative secondary displacement of fractures [12]. In the current retrospective case series study, transverse patellar fractures were fixed with a combination of cannulated screws and the modified Pyrford technique with nonabsorbable polyester sutures to achieve sufficient stable fixation and a lower rate of device removal. The purpose of the current retrospective case series study was to assess and report the clinical outcomes and complications rate of this combined fixation technique.

## Patients and methods

This retrospective clinical study was approved by the medical ethics board of our medical institute. Written informed consent was obtained from all patients. The indications for patellar fractures were as follows: congruity of the patellar surface > 2 mm, a gap between the patellar fragments > 3 mm, or inability to extend the knee due to tearing of the extensor retinaculum. Between January 2015 and February 2021, 94 displaced transverse (OTA/AO 34C1) patellar fractures were confirmed by radiography in our emergency room. All these fractures were surgically treated in our institute.

**Fig. 1:**
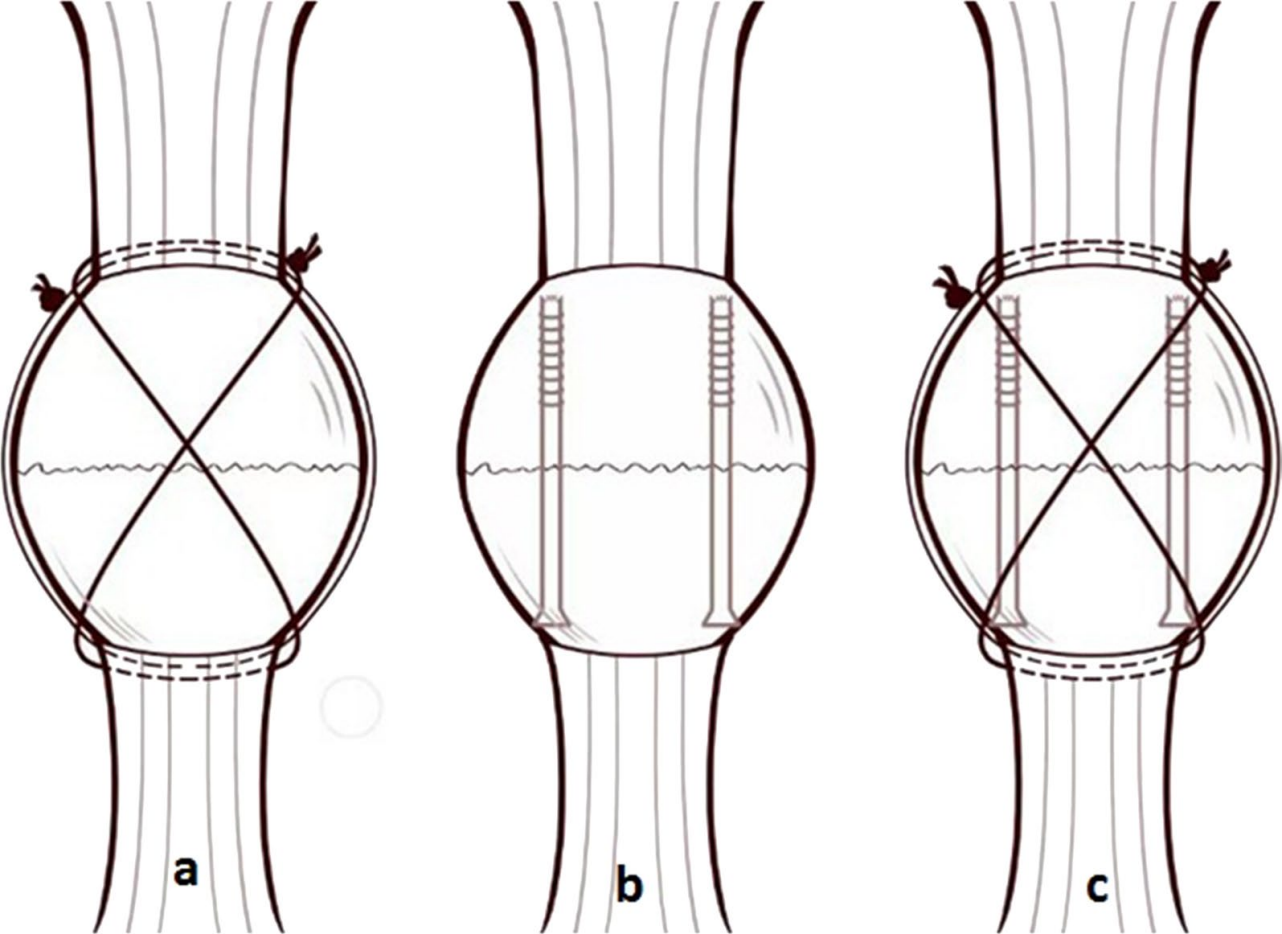
Combined fixation with cannulated screws and the modified Pyrford technique. **a** Modified Pyrford technique; **b** cannulated screw fixation; **c** combined fixation

**Fig. 2:**
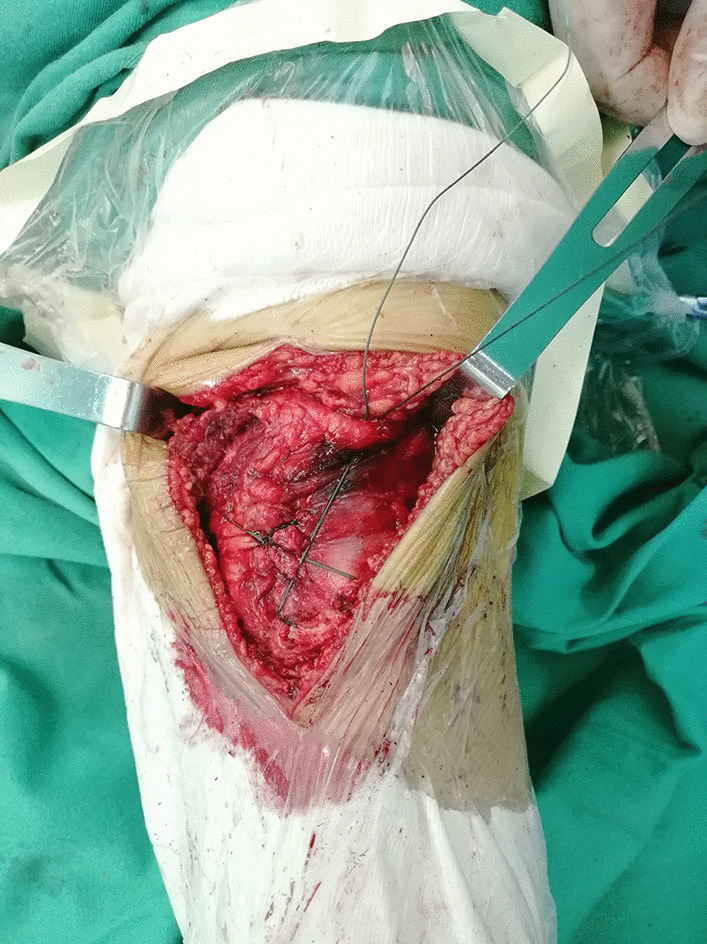
The image of a transverse patellar fracture fixed with combined fixation during the surgery

The patients who underwent the combined fixation technique were placed in the supine position and a tourniquet was applied under anesthesia. An incision was made along the midline of the patella. After exposure of the fracture line, the hematoma in the fracture gap was removed. Then, several irrigations were performed to remove bone debris and fluid from the knee joint cavity. The anterior aponeurosis on the patella within 2 mm of the fracture line was elevated for exposure of the fracture line during reduction. Tight suture of the aponeurosis on the patellar surface was performed to facilitate and simplify the anatomic reduction. The reduction was checked to ensure it was satisfactory, and then temporary fixation was performed with a reduction clamp. After implantation of two longitudinal Kirscher wires, reaming in the smaller fragment was performed along the Kirscher wires in a monocortical fashion. Then, two partially threaded cannulated screws with a diameter of 4.0 mm were inserted. The length of each screw was shorter than the measurement to ensure that the tip of the screw would not protrude from the cortical bone or cut the suture. Then, the cerclage and tension band wiring with nonabsorbable polyester sutures (5 Ethibond-Ethicon Ltd., Edinburgh, UK) were performed in accordance with the Pyrford technique. Importantly, the knot of the Ethibond suture for the cerclage was left on the lateral border of the quadriceps attachment to the patella. The knot of the Ethhibond suture for the tension band was left on the medial border of the quadriceps attachment to the patella. During suturing, the knee joint was kept in extension. Every stitch was pulled forcefully and as close to the patella as possible. When suturing was completed, the stability of the fracture fixation was evaluated several times by full rotation of the knee joint; this was recorded in the surgical record and the physiotherapist was notified to inform individual rehabilitation. Before wound closure, the retinaculum was repaired, and the holes caused by reaming were sutured (see Figs. 1 and 2).

Postoperative rehabilitation was similar for all patients. Each patient had a cast applied with their leg in full extension for wound healing. Isometric quadriceps exercises and partial weight-bearing with crutches were encouraged immediately after surgery. The cast and stitches were removed simultaneously. Thereafter, active and passive knee flexion of 20° or 30°, depending on the stability of the fixation, was instructed. If needed, the affected knee was supported by an adjustable knee brace. With tolerable pain, active knee flexion gradually increased 30° every 2 to 3 weeks after progressive knee flexion training. At 8 weeks postsurgery, the knee brace was removed, and the full range of knee motion was allowed. Importantly, knee rehabilitation was supervised by a physiotherapist to avoid secondary displacement of the fracture or failure of fixation. When bone union was confirmed by radiological examination, full weight-bearing without crutches was allowed.

The electronic medical records of all included patients were reviewed. The collected preoperative data included body mass index (BMI), age, diagnosis of diabetes mellitus, sex, cigarette use, mechanism of injury, and interval between injury and surgery. The included patients were followed up at 1, 2, 3, 6, and 12 months postsurgery, at a minimum. At each follow-up visit, plain radiographs were taken to assess the bone union time and postoperative complications. Furthermore, the requirement for secondary surgery was recorded. The clinical evidence of patellar bone union included no tenderness with local palpation and the ability to continuously walk for 3 min without the aid of a crutch. The radiological evidence of patellar bone union included skeletal trabecula across the fracture line. Delayed bone union was defined as clinical and radiological evidence of bone union between 3 and 6 months postsurgery. If the patient experienced delayed bone union, they were required to attend follow-up appointments every month until bone union was observed. The plain radiographs for all patients were assessed by two orthopedic surgeons. At the 12-month postoperative follow-up, the range of motion (ROM) of the affected knee joint was recorded and clinical outcomes were evaluated by the Bostman scoring system.

## Results

Ninety-four patients were surgically treated for transverse patellar fractures at our institute during the study period. After application of the exclusion criteria, 68 participants were excluded, as follows: lost to follow-up before the 12 months postoperative follow-up (n = 2); impaired extension or flexion function of the ipsilateral knee joint before surgery (n = 2); unable to undergo rehabilitation at the medical instruction or unable to complete the final function evaluation due psychopathy or brain injury (n = 4); presence of an open patellar fracture (n = 1); previous fracture surgically treated on the ipsilateral lower extremity (n = 3); presence of a pathological patellar fracture (n = 0); aged less than 18 years (n = 1); treated with a technique other than the novel combined fixation technique described in this study (n = 47); presence of an old fracture (more than 14 days between the fracture and the surgery) (n = 3); did not provide written informed consent (n = 2); presence of a concomitant fracture or concomitant neurovascular injury on the ipsilateral lower extremity (n = 3). Thus, in total, 26 adult patients diagnosed with transverse patellar fractures treated with this combined fixation technique were included in this study. All surgeries were performed by one experienced orthopedic surgeon.

In the current study, 15 out of 26 patients were female. The average age of the patients was 55.5 ± 20.6 years. Twenty-one patellar fractures were caused by falling, two were caused by sports, and three were caused by traffic accidents. The average BMI of the patients was 24.5 ± 3.1. Out of the 26 patients, five were diagnosed with diabetes mellitus and four had a history of smoking before surgery. The average interval between the injury and surgery was 4.1 ± 1.6 days (see Table 1).

**Table 1:** The demographic data, interval between injury and surgery of the patients

Number	Gender	Age range (years)	Injury mechanism	BMI (kg^2^/m)	Diabetes mellitus	Cigarette use	Interval between injury and surgery (day)
1	Male	16–20	Sports	22.4	No	No	3
2	Female	61–65	Fall	25.1	No	No	5
3	Female	31–35	Fall	18.7	No	No	7
4	Male	21–25	Traffic accident	23	No	No	1
5	Female	66–70	Fall	26.8	No	No	2
6	Female	71–75	Fall	23.6	No	No	5
7	Female	46–50	Fall	21.4	No	Yes	4
8	Male	71–75	Fall	25.5	No	Yes	4
9	Female	56–60	Traffic accident	18.2	No	No	4
10	Female	71–75	Fall	27.9	Yes	No	3
11	Male	46–50	Fall	29.5	No	No	6
12	Female	76–80	Fall	24.4	No	No	3
13	Male	41–45	Fall	24.7	No	No	2
14	Male	26–30	Sports	23.2	No	No	3
15	Female	81–85	Fall	27.9	No	No	5
16	Male	36–40	Fall	27.8	No	No	7
17	Female	26–30	Fall	17.4	No	No	5
18	Male	16–20	Traffic accident	22.6	No	Yes	5
19	Male	51–55	Fall	28.8	Yes	Yes	3
20	Female	76–80	Fall	22.9	No	No	7
21	Male	71–75	Fall	26.3	Yes	No	2
22	Female	76–80	Fall	25.3	Yes	No	4
23	Female	66–70	Fall	24.6	No	No	4
24	Male	61–65	Fall	26.1	No	No	5
25	Female	66–70	Fall	26.3	Yes	No	2
26	Female	66–70	Fall	26.8	No	No	5

*BMI* body mass index

**Fig. 3:**
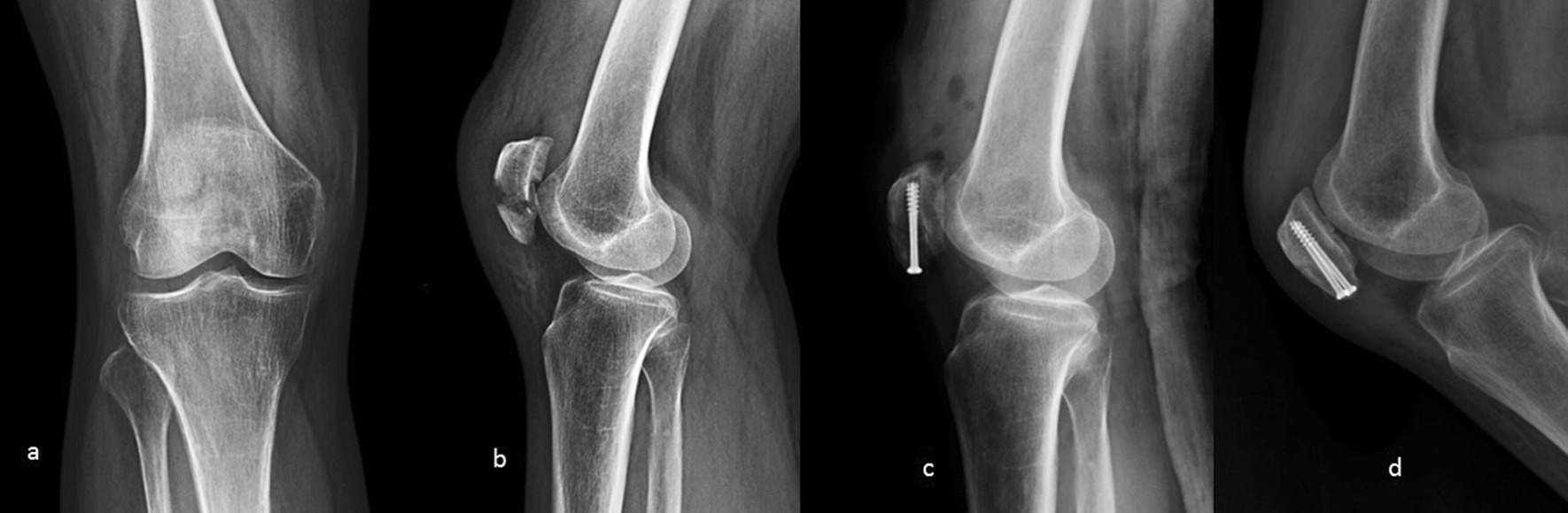
Plain radiographs of the patient (number 12). **a** before surgery; **b** before surgery; **c** at 1 day postsurgery; **d** at 1 year postsurgery

**Fig. 4:**
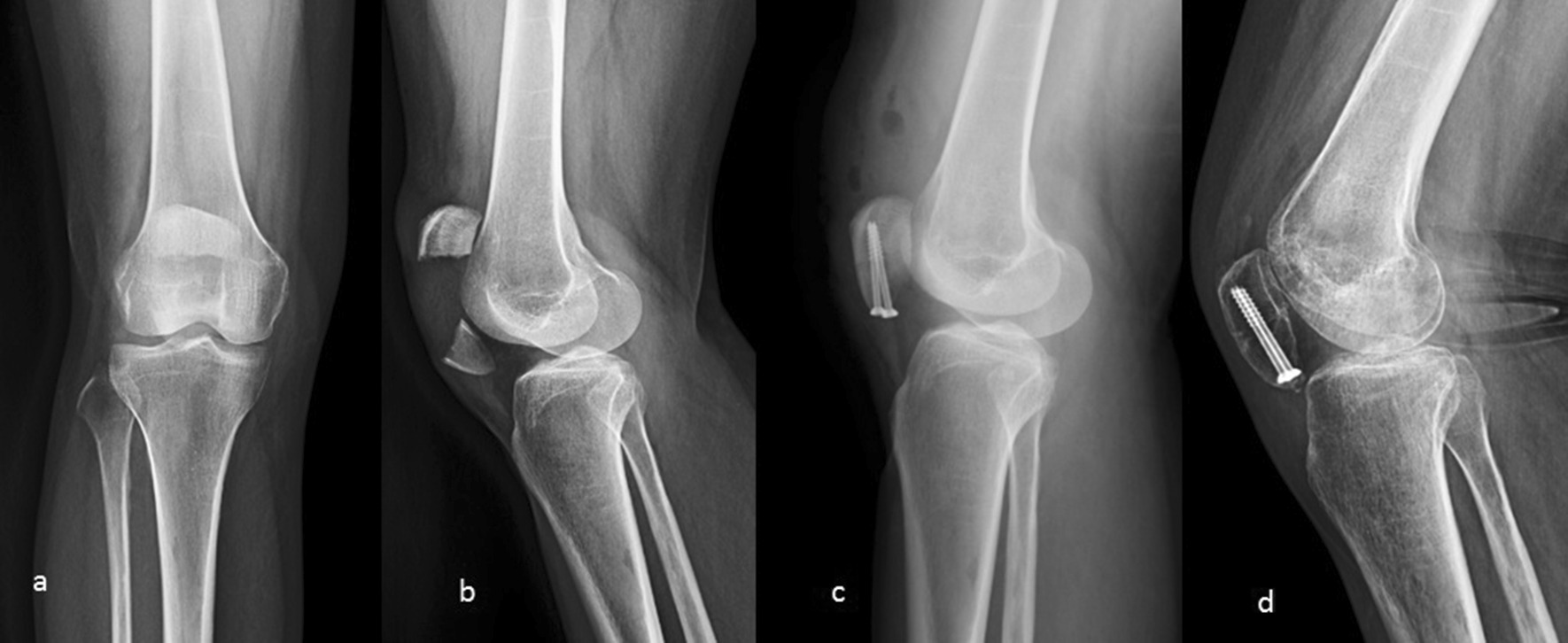
Plain radiographs of the patient (number 23). **a** before surgery; **b** before surgery; **c** immediately after surgery; **d** at 1 year postsurgery

**Fig. 5:**
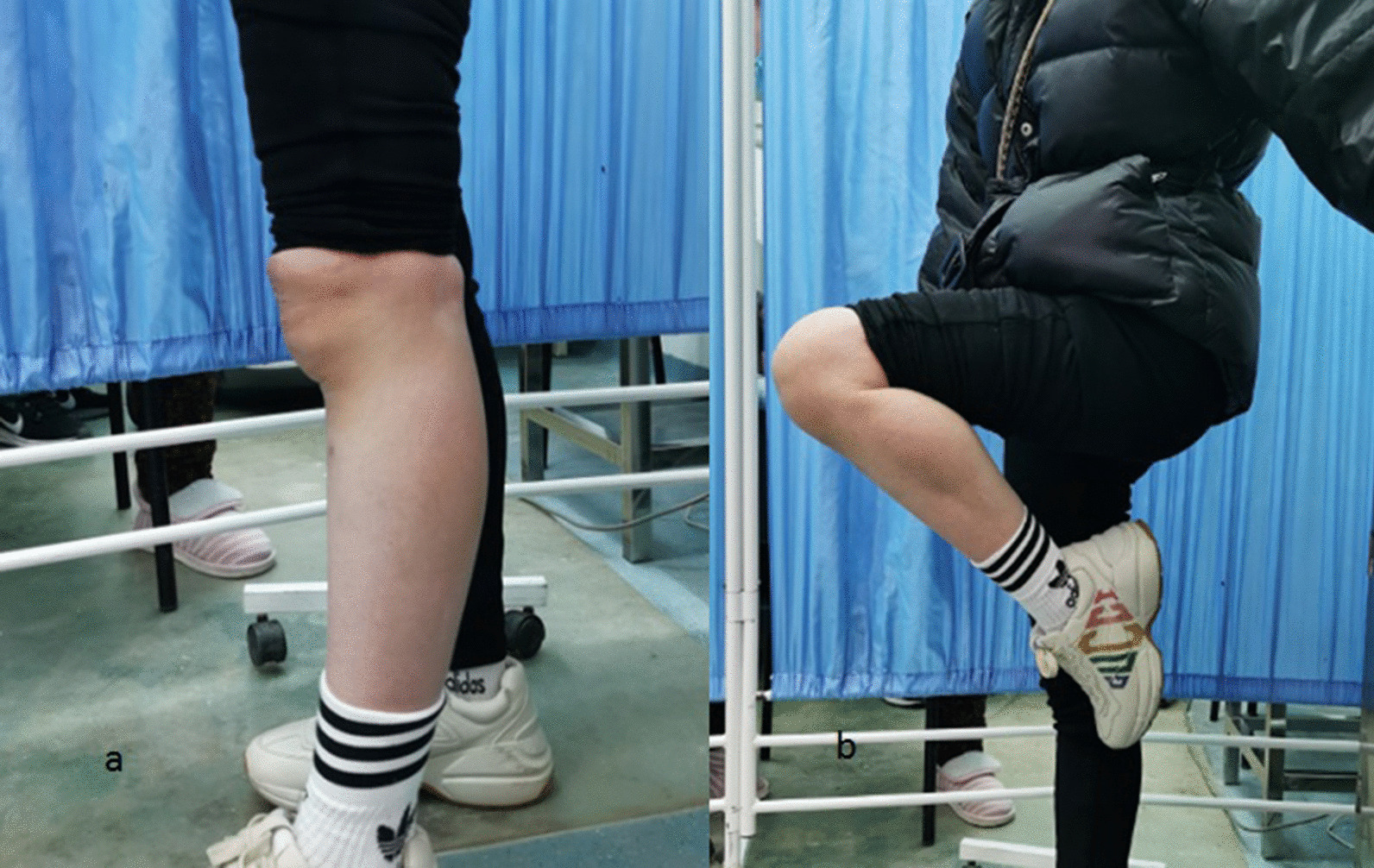
The patient showed the range of motion of the affected knee joint at the final follow-up. **a** Extension; **b** flexion

The average bone union time was 3.0 ± 0.34 months. The follow-up period ranged from 12 to 27 months. Bostman scores were graded into three levels: unsatisfactory (less than 20 points); good (27 to 20 points); excellent (30 to 28 points). The average Bostman score in the current study was 28.3 ± 1.5 (excellent, n = 21, 80.8%; good, n = 5, 19.2%; unsatisfactory, n = 0, 0%) [15]. The average extension and flexion of the knee joint were 1.2 ± 2.1 and 125.6 ± 6.7 degrees, respectively (see Figs. 3, 4 and 5).

Among the 26 patients, one patient (Number 17) experienced delayed bone union; this patient had preoperative chronic kidney disorder and hypoalbuminemia, and bone union was achieved 4 months postsurgery. Another patient (Number 19) experienced abrasion of the patella and was diagnosed with a superficial wound infection; this resolved after treatment with oral antibiotics and removal of the stitches. There were no failed fixations, secondary displacement of the fracture, migrated cannulated screws, or soft tissue irritation in the current study. One patient (Number 4) experienced no postoperative discomfort but required removal of the device for social-psychological reasons. For this patient, removal of the device was performed 1 year postsurgery; no further complications occurred during the later follow-up period (see Table 2).

**Table 2:** The data of follow-up periods, functional outcomes, complications and the requirement for secondary surgeries

Number	Bone healing time (month)	Follow-up periods (month)	Bostman score (points, grading)	Range of motion (extension–flexion, degree)	Complications	Requirement for secondary surgeries
1	2	12	30, Excellent	5–135	No	No
2	3	16	30, Excellent	5–125	No	No
3	3	14	29, Excellent	5–135	No	No
4	3	18	29, Excellent	5–135	No	No
5	3	15	28, Excellent	5–125	No	No
6	3	27	26, Good	0–120	Pain after long distance walking or stair-climbing	No
7	3	18	28, Excellent	5–125	No	No
8	3	16	28, Excellent	0–125	No	No
9	3	12	28, Excellent	0–130	No	No
10	3	19	29, Excellent	5–125	No	No
11	3	13	29, Excellent	5–125	No	No
12	3	18	28, Excellent	0–125	No	No
13	3	16	30, Excellent	5–130	No	No
14	2	12	30, Excellent	5–135	No	No
15	3	26	25, Good	0–110	Pain after long distance walking or stair-climbing	No
16	3	12	30, Excellent	5–130	No	No
17	4	16	29, Excellent	0–125	Delayed bone healing	No
18	3	24	28, Excellent	0–130	No	removal
19	3	17	28, Excellent	0–130	Superficial infection	No
20	3	12	28, Excellent	0–125	No	No
21	3	14	28, Excellent	0–125	No	No
22	3	19	26, Good	0–110	No	No
23	3	15	28, Excellent	0–125	No	No
24	3	12	29, Excellent	5–125	No	No
25	3	23	27, Good	0–105	Pain after long distance walking or stair-climbing	No
26	3	12	30, Excellent	5–125	No	No

Bostman score: excellent, 30 to 28 points; good, 27 to 20 points; unsatisfactory, < 20 points

## Discussion

Transverse fractures of the patella are the most common type of patellar fracture [16]. In a cadaveric biomechanical study, Carpenter et al. [17] analyzed the stability of transverse patellar fractures fixed with three different techniques: modified tension bands, two parallel interfragmentary lag screws, and two cannulated lag screws with a tension band wire through the middle of the cannulated screws. The authors reported significantly better stability after fixation with cannulated screws and a tension band wire. Therefore, this technique was regarded as the “gold standard” for treating noncomminuted transverse patellar fractures. However, Hoshino et al. found that 30 out of 133 (22.6%) patients who received surgical treatment of transverse patellar fractures with metallic tension bands through double cannulated lag screws had to undergo secondary surgery to remove the device due to device irritation or wound infection [9]. In China, Buddhism is the most popular religion [18]. For Buddhists, the daily religious ritual of kneeling makes device irritation intolerable and results in a strong desire for device removal. The results of a systematic review conducted by Camarda et al. in 2016 indicated that fixation of patellar fractures with nonmetallic devices is associated with a lower secondary surgery rate (1.6%) for device removal [19]. In biomechanical studies [20, 21], the ultimate failure strength of No. 5 Ethibond was reported to be 207.4 N, which was obviously lower than the ultimate failure strength of 18 gauge stainless steel wire (910 N). In 2019, Westberg et al. [22] evaluated and compared the biomechanical properties of the cerclage fixation with No.5 Ethibond sutures and the cerclage fixation with 18 gauge stainless steel wire. They [22] found that the ultimate failure strength of cerclage fixation with No.5 Ethibond suture was significantly higher than the fixation with 18 gauge stainless steel wire. Moreover, Patel et al. [23] reported that fixation with nonabsorbable polyester sutures in place of 18 gauge wire is a satisfactory surgical treatment for patellar fractures as the strength of nonabsorbable polyester sutures is considered to be sufficient for postoperative rehabilitation. Gosal et al. [10] fixed 16 patellar fractures with nonabsorbable polyester sutures in accordance with the modified Pyrford technique and reported one fixation failure (6.3%). Similarly, Egol et al. [13] fixed patellar fractures in a Krackow-type fashion with nonabsorbable sutures and reported an initial failure rate of 7.6%. Illical et al. [7] conducted a randomized controlled study to compare the functional outcomes and complications of patients with patellar fractures fixed with double Kirscher wires and tension bands with either stainless steel wire or Ethibond sutures. The authors reported that no postoperative secondary surgery was required due to failure of fixation in the Ethibond group, and the European Quality of Life-5 Dimensions score in the Ethibond group was significantly better than that of the steel wire group [7]. Therefore, increasing the strength of the low-profile construct of sutures seems essential for treating patellar fractures and achieving satisfactory clinical outcomes as well as lower rates of complications and secondary surgeries.

In the current study, transverse patellar fractures were surgically treated with two cannulated screws plus the modified Pyrford technique with polyester sutures. No cases of postoperative failure of fixation or secondary displacement of fractures were observed. In the biomechanical study conducted by Carpenter et al. [17], the load to failure of transverse patellar fractures after fixation with screws only was lower than that after screws with a tension band; however, the difference was not significant (p = 0.06). Due to the better stability of transverse patellar fractures compared with comminuted patellar fractures, Gwinner et al. [24] reported that screw fixation without a tension band can be considered for such fracture types, although this technique is weaker than the use of cannulated screws with metallic tension band wire. Given the high level of stiffness and minimal tissue reaction to polyester sutures, Qi et al. fixed patellar fractures with polyester suture tension bands through two cannulated screws and reported a mean Lysholm score of 95.7 [25–27]. In their study, Qi et al. [27] found no failure of fixation, secondary displacement, infection, or device migration or breakage. Similarly, Busel et al. [28] conducted a retrospective case series analysis in 2019 and found that the secondary surgery rate for device removal after fixation with nonabsorbable sutures through cannulated lag screws was 8%; all device removals were due to hardware irritations. However, Kumar et al. [29] reported that the tension band technique with nonabsorbable polyester sutures through cannulated lag screws is difficult, and even impossible, to perform when using screws with a small middle diameter. In such cases, the thickness of the needle required to hold the suture and the thickness of the folded suture when passing the suture through the cannulated screws is usually too large for the middle of the screws. As an alternative to polyester suture tension bands through cannulated screws, a variety of fixation techniques using nonabsorbable polyester sutures have been introduced for treating patellar fractures; these techniques provide sufficient stability for early rehabilitation after surgery [12, 30–33]. To date, there has been no consensus regarding the best fixation technique with nonabsorbable sutures for the treatment of patellar fractures. Camarda et al. [12] fixed patellar fractures with FiberWire following the Pyrford technique and reported a high rate of ‘excellent’ Bostman scores (76.4%) and an absence of failure of fixation. However, 11.8% of patients experienced secondary displacement of fractures [12]. Furthermore, this study defined secondary displacement as a fracture gap less than 4 mm, which might be larger than the indication for surgery (gap > 3 mm) in the current study [12]. In the biomechanical study conducted by Burvant et al. [34], the displacement rate of fractures fixed with screws plus the Pyrford technique was dramatically smaller than the displacement rate of fractures fixed with the Pyrford technique only. Therefore, the superiority of the combined technique in this study over the modified Pyrford technique may be due to the more stable fixation.

In terms of the Bostman scores and gradings, our findings are consistent with those after fixation with cannulated screws and metallic tension bands as reported by Tan et al. [35] and with those after fixation with the modified Pyrford technique as reported by Camarda et al. [12]. The ROM in the current study was comparable, although somewhat smaller, than the ROM reported in previous studies that used tension band fixation with suture materials [12, 27, 36]. This is perhaps due to the concern of less stable fixation without metallic wires and subsequent individual rehabilitation. In the current study, the postoperative infection rate was 3.8%, which is consistent with the postoperative infection rate of 3.6% after metallic tension band wire fixation for patellar fractures reported by Hoshino et al. [27]. However, the infection in the current study was resolved by oral antibiotics and no further surgery was needed. In the clinical study conducted by Hoshino et al. [27], 43.8% of the infections required intravenous antibiotics administration or further surgical interference.

The relatively small sample size is the main limitation of this study. Thus, there is a need for the treatment of more cases with the combined technique in order to improve the reliability of the research. Because of the absence of inclusion criteria in this case series study, the fixation technique decision for each patient was made by the surgeons, resulting in selection bias. Furthermore, the nature of a case series study means that this study lacked a control group and may not be as reliable as a case–control study. This study may have also been affected by recall biases. Therefore, the conclusions of this study should be confirmed by a prospective randomized controlled study in the future.

## Conclusions

Our combined technique for fixation of transverse patellar fractures with cannulated screws and the modified Pyrford technique using suture materials had excellent clinical outcomes and a low rate of complications. This technique may be considered a better treatment approach for common transverse patellar fractures due to the lower rate of device removal.

## Data Availability

The data supporting the conclusions of the current study are included within the article, tables and figures. The datasets generated and/or analysed during the current study are not publicly available due to restrictions on ethical approvals involving patient data and anonymity but are available from the corresponding author on reasonable request.

## Abbreviations

OTA/AO: Orthopaedic Trauma Association/Arbeitsgemeinschaft für Osteosythese
BMI: Body mass index
ROM: Range of motion
